# The Forkhead Transcription Factor Foxl2 Is Sumoylated in Both Human and Mouse: Sumoylation Affects Its Stability, Localization, and Activity

**DOI:** 10.1371/journal.pone.0009477

**Published:** 2010-03-02

**Authors:** Mara Marongiu, Manila Deiana, Alessandra Meloni, Loredana Marcia, Alessandro Puddu, Antonio Cao, David Schlessinger, Laura Crisponi

**Affiliations:** 1 Istituto di Neurogenetica e Neurofarmacologia, Consiglio Nazionale delle Ricerche, Cagliari, Italy; 2 Università degli Studi di Cagliari, Cagliari, Italy; 3 National Institute on Aging, National Institiutes of Health, Baltimore, Maryland, United States of America; George Washington University, United States of America

## Abstract

The FOXL2 forkhead transcription factor is expressed in ovarian granulosa cells, and mutated *FOXL2* causes the blepharophimosis, ptosis and epicanthus inversus syndrome (BPES) and predisposes to premature ovarian failure. Inactivation of *Foxl2* in mice demonstrated its indispensability for female gonadal sex determination and ovary development and revealed its antagonism of Sox9, the effector of male testis development. To help to define the regulatory activities of FOXL2, we looked for interacting proteins. Based on yeast two-hybrid screening, we found that FOXL2 interacts with PIAS1 and UBC9, both parts of the sumoylation machinery. We showed that human FOXL2 is sumoylated in transfected cell lines, and that endogenous mouse Foxl2 is comparably sumoylated. This modification changes its cellular localization, stability and transcriptional activity. It is intriguing that similar sumoylation and regulatory consequences have also been reported for SOX9, the male counterpart of FOXL2 in somatic gonadal tissues.

## Introduction

Mutations in *FOXL2* cause the blepharophimosis, ptosis and epicanthus inversus syndrome (BPES MIM 110100) either with (Type I) or without (Type II) premature ovarian failure [1]. We created a mouse model for BPES through the disruption of *Foxl2*, and showed that *Foxl2* is required for ovarian follicle formation [2]. The failure of Foxl2-negative granulosa cells to sustain follicle formation is a primary cause of ovarian failure, leading to subsequent deregulated oogenesis by an unknown mechanism [2]. In addition to its role in follicle formation and oocyte growth dynamics, we demonstrated an apparent role for FOXL2 in repressing male sex determination. A male differentiation program is initiated in *Foxl2^−/−^* female ovaries, with *Sox9*, the master effector of gonadal testis differentiation, sharply upregulated between birth and 1 week postnatum [3]. During this transition, *Foxl2*-null ovaries contain cords, delimited by basal lamina, that enclose cells with male Sertoli-like features. Based on these observations and supporting correlated global gene expression changes, we have suggested that mammalian female sex determination is labile and may require Foxl2 activity in the gonadal soma throughout ovary development and maturation [3]. The data are consistent with sex determination occurring in the bipotential gonad with a direct parallelism between two intrinsically antagonistic transcription factors, Foxl2 and Sox9, one with a key role in female and the other in male mammalian gonadal development [4]. In fact, *SOX9* heterozygous mutations cause the severe campomelic dysplasia syndrome (CMPD MIM 114290), in which - in addition to defects in cartilage and bone - about 75% of affected XY individuals have a sex-reversed phenotype [5].

Here we provide evidence that the antagonists Foxl2 and Sox9 also show a parallel modulation of their activity by sumoylation. As described for SOX9 [6], [7], we found that FOXL2 interacts with PIAS1 and is sumoylated. Sumoylation is a highly dynamic and reversible process, exerting pleiotropic biological effects that include changes in subcellular localization, protein partnering and modulation of the DNA binding and activity of transcription factors, which are the largest group of target proteins affected by sumoylation [8], [9]. In the case of FOXL2, we report changes in its localization, stability and activity.

## Methods

### Ethics Statement

Mice were manipulated and housed according to the European Community Council Directive (EEC/609/86) and to the Italian guidelines DL 116/1992. The experimental protocol and the detailed application form that focuses on how the animals have been used, has been approved by Italy's National Institute of Health, in particular by the Service for Biotechnology and Animal Welfare, and by University of Cagliari.

### Mice

C57BL/6 female mice were obtained from Charles River laboratory (Calco - Lecco, Italy). Mice were housed conventionally in a constant temperature (20–24°C) and humidity (50–60%) animal room and with a 12 h light–dark cycle. All mice had free access to food and water. Mice were sacrificed at 4 weeks of age by CO2 asphyxiation or cervical dislocation.

### Yeast Two Hybrid Assay

A yeast two-hybrid screen was performed using the Matchmaker™ Two-Hybrid System 3 (Clontech). Full-length *FOXL2* cDNA was cloned into pGBKT7 vector, and transformed into AH109 yeast cells. Human Ovary MATCHMAKER cDNA Library (Clontech) cloned in the pACT2 vector was transformed into AH109 cells already containing FOXL2 as a bait, and co-transformants were screened on selection plates according to the Clontech protocol. False positives were reduced by performing an X-alpha-galactosidase assay.

### Cell Culture, DNA Transfections, Cell Lysis

COS-7 cells (ATCC) were grown in DMEM (Dulbecco's Modified Eagle's Medium) supplemented with 10% fetal bovine serum, 100 U/ml penicillin, 100 µg/ml streptomycin and 2 mM glutamine. COS-7 cells were transfected using Lipofectamine 2000 (Invitrogen) and after 48 h were lysed with modified RIPA buffer (140 mM NaCl, 10 mM TrisHCl pH 7.5, 1 mM EDTA, 1% Triton x-100, 0.1% Sodium deoxycholate) containing Complete Protease Inhibitor Cocktail (Roche) and 40 mM of N-ethylmaleimide (NEM), according to Hattori et al. 2006 (6). After brief sonication and 30 min on ice, the lysates were centrifuged at 4°C, 16000×g, for 20 min. Protein concentrations were measured by Bio-Rad Protein Assay. For half-life determination and stability assays, Cycloheximide (Sigma-Aldrich, C7698) at final concentration 100 µg/ml, and MG132 (Calbiochem) at final concentration 10^−8^ M were used for the indicated times. Alpha T31 pituitary mouse cell line (α-T31) were kindly donated by P.L. Mellon (Department of Reproductive Medicine, University of California, San Diego, USA) and were grown in RPMI 1640 supplemented with 10% fetal bovine serum, 100 U/ml penicillin, 100 µg/ml streptomycin and 2 mM glutamine.

### Constructs, Immunoblotting and Immunoprecipitation

Human full length *PIAS1* and *UBC9* cDNA obtained from pACT2 vectors isolated by two hybrid screen, and *FOXL2* were cloned into the pCRUZ-HA or pCRUZ-myc vectors (Santa Cruz). Full length *SUMO-1* cDNA was derived from a pGEX2T-SUMO-1 vector kindly provided by Ronald T. Hay (Wellcome Biocentre, University of Dundee, U.K.), and cloned into the pCRUZ-HA expression vector. For immunoprecipitation, the lysates were diluted 4-fold in co-immunoprecipitation buffer (2 mM Hepes pH 7.9, 20 mM NaCl, 0,01% Tween 20, 1 mM DTT, 10% glycerol) and incubated overnight with 25 µl of anti-myc (Santa Cruz) or anti-FOXL2 agarose-conjugate antibody at 4°C. The immunocomplexes were washed 5 times with 800 µl of co-immunoprecipitation buffer, and immunoprecipitated proteins were released from the antibody-agarose-conjugate by the addition of 25 µl of glycine pH 2.5 with a 2 min incubation in ice. Samples were fractionated on a 7.5% SDS-polyacrylamide gel, then transferred to PVDF membranes (HYBOND-P, Amersham Biosciences), hybridized with the primary antibody overnight, then with a horseradish peroxidase-conjugated secondary antibody that was detected by enhanced chemiluminescence (ECL, Amersham Biosciences). The primary antibodies used were mouse anti-myc (Santa Cruz sc40, 1∶200), rabbit anti-HA (Santa Cruz sc805, 1∶200), rabbit anti-SUMO-1 (Santa Cruz sc9060, 1∶500), rabbit anti-UBC9 (Santa Cruz sc10759, 1∶400) rabbit anti-PIAS1 (Santa Cruz sc14016 1∶500) rabbit anti-ubiquitin (Sigma-Aldrich U5379, 1∶500) and rabbit polyclonal anti-FOXL2 raised against residues DHDSKTGALHSRLDL (Eurogentec s.a., Belgium) and used after affinity purification. Secondary antibodies were purchased by Santa Cruz biotechnology inc, goat anti-mouse IgG (Santa Cruz sc2005, 1∶100,000), goat anti-rabbit IgG (Santa Cruz sc2004, 1∶100,000). Densitometric analysis to evaluate the intensities of protein bands was performed by Image J software (http://rsbweb.nih.gov/ij/).

### Site-Directed Mutagenesis

FOXL2 mutants FOXL2-K25R, FOXL2-K87R, FOXL2-K114R and FOXL2-K150R, single or all 4 together (FOXL2-KFULL), were generated in the pCRUZ-myc FOXL2 vector using the QuickChange Site-Directed Mutagenesis kit (Stratagene) according to the manufacturer's protocol. Primer sequences are available on request.

### Immunofluorescence and Confocal Microscopy

4 week ovaries isolated from C57BL/6 mice were fixed in Histochoice (Amresco, Solon, OH, USA) at room temperature for 4 h. Sections were treated with 3% H_2_O_2_ for 1 h and unmasked with Citrate Buffer solution (LAB VISION corp. AP-9003-500) and with 0.01 M EDTA, pH 8. Slides were blocked with DakoCytomation Protein Block Serum-free (X0909) for 30 min at room temperature and incubated overnight at 4°C with primary antibodies. Primary antibody dilutions were as follows: anti-FOXL2 goat polyclonal (ABCAM ab5096, 1∶200), anti-SUMO-1 rabbit polyclonal (Santa Cruz sc9060, 1∶25), anti-UBC9 rabbit (Santa Cruz sc10759, 1∶100). COS-7 cells were co-transfected either with pCRUZ-myc FOXL2 wild type or mutated forms and pCRUZ-HA SUMO-1 for 48 h. Cells fixed at room temperature for 1 h in Histochoice were unmasked in Citrate Buffer solution for 20 sec in a microwave, permeabilized with 20 mM Tris-HCl, pH 7.6, 137 mM NaCl, and 0.1% Tween 20 containing 5% skim milk for 30 min at room temperature (according to Hattori et al., ref. 6) and incubated with primary antibodies overnight at 4°C. Alexa Fluor 594 anti-goat, Alexa Fluor 633 anti-mouse, and Alexa Fluor 488 anti-rabbit secondary antibodies (Molecular Probes) were diluted 1∶500. Immunofluorescence analysis was performed using a Leica DMIRE2-TCS-SL Confocal Laser Scanning microscope (488 to 633 excitation wavelength); images were acquired with a 40X objective.

### Transactivation Assays

A 972 bp fragment of the human *StAR* promoter was amplified by PCR from genomic DNA using the following primers: forward 5′ ctctcgcgagagggtggtt 3′ and reverse 5′ gcttgaatgtcgctagcagc 3′. This PCR product was digested with NruI and HindIII and cloned into the plasmid vector pGL3 basic (Promega). COS-7 cells were transfected using Lipofectamine 2000 (Invitrogen) with 1 µg of human pCRUZ-myc FOXL2, 1 µg of human *StAR* promoter luciferase construct, and 4 µg of human pCRUZ-HA SUMO-1. A Renilla luciferase control vector (Promega) was co-transfected to normalize data. After 48 h transfected cells were treated according to Dual-Luciferase Reporter Assay System (Promega) protocol and luciferase activities was measured. At least 3 independent transfections were carried out and each experiment was performed in quadruplicate. Values are mean +/− SD of at least 4 independent assays. Statistical significance was estimated by Student's t-Test.

## Results

Although the importance of the forkhead transcription factor FOXL2 in ovarian development and function is established, regulation of its action is not well understood. We found that post-translational modification by sumoylation affects both FOXL2 metabolism and activity.

### Forkhead Transcription Factor FOXL2 Interacts with PIAS1 and UBC9, Members of the Sumoylation Machinery

To look for proteins interacting with FOXL2, we initially performed a yeast two hybrid assay to screen a human ovary cDNA library. Both bait plasmid PGBKT7, containing the full-length FOXL2 fused in-frame to the DNA binding domain of Gal4, and a human ovary cDNA library, fused to the Gal4 activator domain in PGADT7, were introduced into AH109 yeast cells. A total of 4×10^6^ independent transformants were screened for the expression of four reporter genes (*ADE2*, *HIS3*, *MEL*, and *lacZ*). Among 288 colonies that tested positive, DNA sequencing identified 8 *PIAS1* clones and 69 *UBC9* clones. In controls, co-transformation of empty prey plasmid with a FOXL2, PIAS1, or UBC9 bait plasmid failed to activate expression of reporter genes.

PIAS1 and UBC9 are both tell-tale members of the sumoylation machinery. In outline, sumoylation involves the reversible covalent attachment of a SUMO protein moiety (about 11 kDa) to lysine residues in proteins through a three-step process, engaging the E1-activating molecules SAE1/SAE2, the E2-conjugating enzyme UBC9, and E3 ligases such as PIAS in mammalian cells that confer specificity [8], [9].

**Figure 1:**
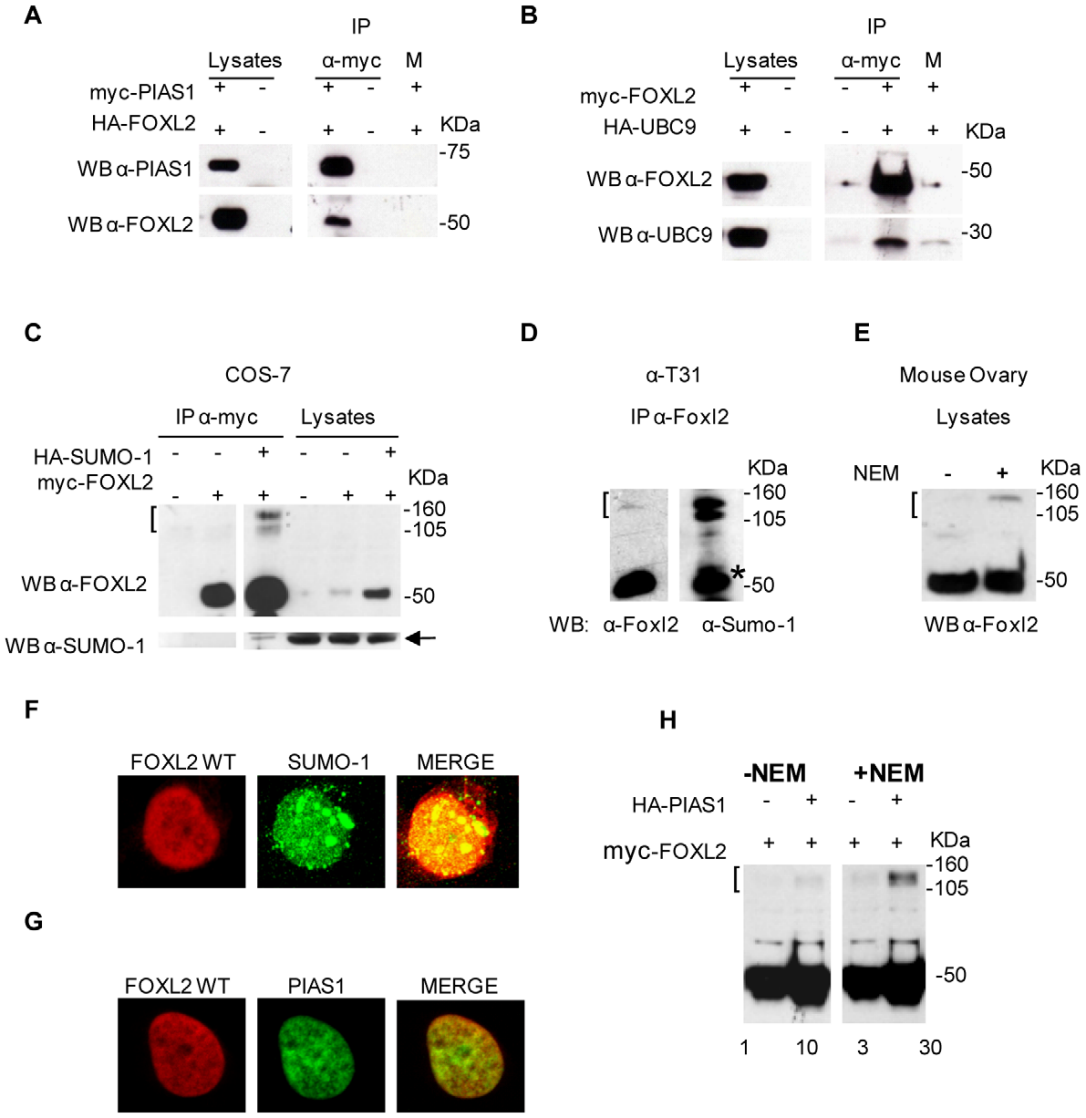
FOXL2 interacts with sumoylation machinery, is sumoylated and co-localize with SUMO-1 and PIAS1. *A., B.* FOXL2 interacts with PIAS1 and UBC9: COS-7 cells were co-transfected with pCRUZ-HA-FOXL2 and pCRUZ-myc-PIAS1 (A), or with pCRUZ-myc-FOXL2 and pCRUZ-HA-UBC9 (B) or with HA and myc empty vectors (−). Lysates were immunoprecipitated with anti-myc agarose conjugated antibody and analysed by western blotting with anti-FOXL2, anti-PIAS1 and anti-UBC9 antibodies. Mock IP consisted in an immunoprecipitation with only protein-A beads, without antibody. Expression of all proteins was also analysed in total lysates (input). *C.* FOXL2 is sumoylated in transfected COS-7 cells: COS-7 cells were transfected with pCRUZ-myc-FOXL2 (0.5 µg) alone or with pCRUZ-HA-SUMO-1 (4.5 µg), or with HA and myc empty vectors (−). Lysates were immunoprecipitated with anti-myc agarose conjugated antibody, and analysed by western blotting using anti-FOXL2 antibody (upper panel) or anti-SUMO-1 antibody (lower panel). Both antibodies recognized band(s) of about 105-160-kDa indicated by brackets (**[**), not present in the transfection of FOXL2 alone. *D.,E.* FOXL2 is sumoylated *in vivo* in physiological conditions: (D) Immunoprecipitation was done using anti-FOXL2 antibody on α-T31 cell lysate and the eluate analyzed by western blotting using anti-FOXL2 antibody (left panel) or anti-SUMO-1 antibody (right panel). Both antibodies recognized band(s) of about 105–160-kDa indicated by brackets (**[**). The asterisk indicates the FOXL2 signal from the previous hybridization. (E) 4-week old mouse ovaries were lysed with or without NEM, 60 µg of protein was loaded on SDS PAGE, electrophoresed, and then immunoblotted. Western blotting with an anti-Foxl2 antibody showed a 45 kDa band corresponding to native Foxl2 and a slower migrating band of about 105–160 kDa, also recognised by anti-Sumo-1 antibody (not shown). *F.* FOXL2 and SUMO-1 co-localize in the nucleus: pCRUZ-myc-FOXL2 and pCRUZ-HA-SUMO-1 were co-transfected into COS7 cells, and immunofluorescence was performed using anti-myc and anti-HA antibody. In red (Alexa 633) is shown FOXL2, in green (Alexa 488) SUMO-1. The yellow colour indicates co-localization, and is particularly seen in spots resembling PML bodies. *G.* Wild type FOXL2 co-localizes with PIAS1 in the nucleus: COS-7 cells were co-transfected with pCRUZ-myc-FOXL2 and pCRUZ-HA-PIAS1. The intracellular distribution of FOXL2 (red) and PIAS1 (green) was detected by indirect immunofluorescence with mouse anti-myc and rabbit anti-HA primary antibodies and Alexa Fluor 633 anti-mouse and Alexa Fluor 488 anti-rabbit secondary antibodies. *H.* PIAS1 enhances FOXL2 sumoylation: COS-7 cells were co-transfected with pCRUZ-myc-FOXL2 and pCRUZ-HA-PIAS1. And lysed with or without NEM. Lysates were immunoblotted with anti-FOXL2 antibody and densitometric analysis was performed on sumoylated band using Image J software. Densitometric analysis is reported compared to that in lane 1.

The FOXL2 association with PIAS1 and UBC9 was validated by several approaches: 1. Co-transformation in yeast. AH109 yeast strain was co-transformed with the pGBKT7 vector expressing full-length FOXL2 and the pGADT7 vector expressing full-length PIAS1 or UBC9. The empty pGADT7 vector was also re-transformed along with FOXL2 as a control. FOXL2 bound strongly to PIAS1 and also bound, though more weakly, to UBC9; the same interactions were detected when the PIAS1 and UBC9 were switched from the pGADT7 vector to pGBKT7 and FOXL2 from the pGBKT7 to the pGADT7 vector and were re-tested in the two-hybrid assay. The strength of the interactions was determined by quantitative measurement in liquid β-galactosidase assays (data not shown). 2. Co-immunoprecipitation *in vivo*. More direct evidence of interaction was obtained when COS-7 cells were co-transfected with myc-FOXL2 and HA-UBC9 or with myc-PIAS1 and HA-FOXL2 constructs. A protein lysate from the cells was then immunoprecipitated using an anti-myc antibody and analysed by Western blotting with anti-tag and anti-UBC9 or anti-PIAS1 specific antibodies. Binding to FOXL2 was confirmed for both PIAS1 and UBC9 (Fig. 1A, B).

### FOXL2 is Sumoylated and Co-Localizes with SUMO-1

To test whether FOXL2 undergoes sumoylation, we transiently co-transfected monkey kidney COS-7 cell lines with constructs encoding myc-FOXL2 wild type and HA-SUMO-1. Cell lysates were prepared and FOXL2 was immunoprecipitated by anti-myc antibody, then analysed by Western blotting. Anti-FOXL2 antibody detected additional higher molecular weight species of about 105–160 kDa (versus 50 kDa for the native protein) in the presence of SUMO-1. They were missing or reduced in the sample transfected with FOXL2 alone (Fig. 1C). The observations in transfected COS-7 cells were corroborated for the endogenous Foxl2 mouse homolog by immunoprecipitation in α-T31 pituitary cells (Fig. 1D). These higher band(s) were also detected by anti-SUMO-1 antibody (Fig. 1C, D). The 105–160-kDa “poly SUMO-FOXL2” species appear as single or double bands in different experiments, and this could be assessed by antibodies/experimental condition/tag used. Furthermore, 4 week-old mouse ovaries were lysed with and without NEM, which inhibits desumoylases, and the protein extracts were immunoblotted with anti-FOXL2 antibody. Only in the NEM-treated extracts were the higher molecular weight band(s) evident (Fig. 1E). When FOXL2 was co-transfected with SUMO-1 in COS-7 cells, co-localization was observed by confocal microscopy in structures reminiscent of PML (promyelocytic leukaemia) bodies (Fig. 1F). SUMO proteins have been shown to localize to specific subnuclear structures known as PML-containing nuclear bodies, associated with the nuclear matrix [10], [11]. These findings are consistent with sumoylation of FOXL2 *in vivo*. Addition of a single SUMO-1 moiety would add a molecular mass of about 11 kDa, so that the apparent increase of more than 50 kDa in denaturing gels suggests that 3 to 5 SUMO groups might be conjugated on FOXL2 to account for the various species seen and there might be multiple sumoylation sites with a single SUMO-1 moiety.

### PIAS1 Co-Localizes with FOXL2 and Acts as an E3 Ligase for FOXL2 Sumoylation

Sumoylation proceeds via a multi-enzyme pathway. SUMO-1 is activated by an E1 activating enzyme (the heterodimer Aos1/Uba2) and is then transferred to the E2 conjugating enzyme (UBC9), and thence to a specific lysine in the target protein by an a E3 ligase. Proteins of the PIAS family can act as SUMO-3 ligases, significantly enhancing the level of the substrate sumoylation [8]. To determine whether PIAS1 can increase FOXL2 sumoylation, we co-transfected COS-7 cells with myc-FOXL2 and HA-PIAS1, both in the presence or absence of NEM. We found that FOXL2 and PIAS1 co-localize in the nucleus (Fig. 1G); that PIAS1 caused notable enhancement of the 105–160-kDa “poly SUMO-FOXL2” species; and that NEM increased the intensity of these bands (Fig. 1H).

### Does Sumoylation Regulate FOXL2 Stability?

**Figure 2:**
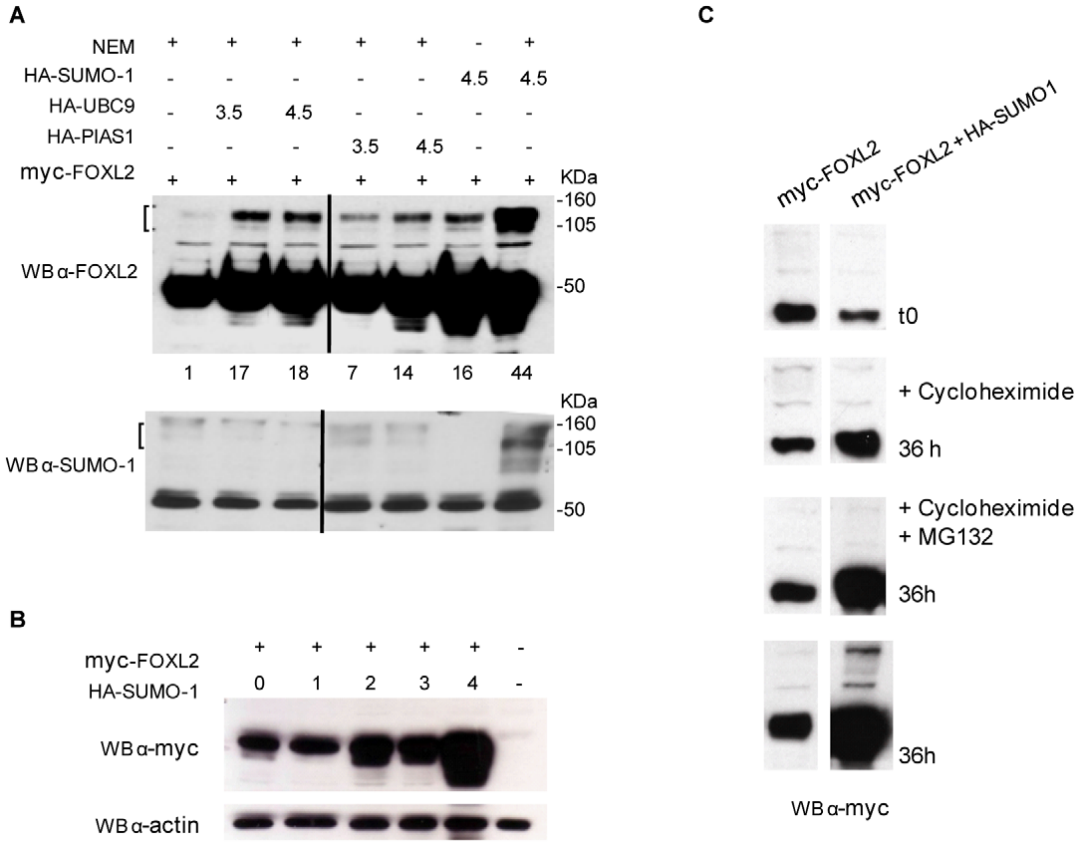
FOXL2 stability is increased by SUMO-1 and is not dependent on proteasome-mediated degradation. *A.* Sumoylation regulates FOXL2 stability: COS-7 cells were co-transfected with pCRUZ-myc-FOXL2 (0.5 µg) and increasing amounts of pCRUZ-HA-UBC9 or pCRUZ-HA-PIAS1(3.5–4.5 µg) or pCRUZ-HA-SUMO-1 (4.5 µg). Lysates treated with and without NEM were analysed by immunoblotting with anti-FOXL2 antibody. The bracket shows slower migrating bands of about 105–160 kDa. Densitometric analysis was performed on sumoylated band using Image J software and is reported compared to that in lane 1. *B.* FOXL2 stabilization is mediated by SUMO-1 in a dose-dependent manner: COS7 were transfected with 500 ng of pCRUZ-myc-FOXL2 and increasing amounts of pCRUZ-HA-SUMO-1 (0, 1, 2, 3, 4 µg). Immunoblotting against myc shows that FOXL2 increases with the augmentation of SUMO-1. In the lower panel anti-actin is used as loading control. *C.* FOXL2 stability is not dependent on ubiquitination and proteasome-mediated degradation: COS7 cells were transfected with pCRUZ-HA-FOXL2 alone and with pCRUZ-HA-SUMO-1 in quadruplicate. After 12 h of transfection, cells were lysed (t0) or treated with cycloheximide (inhibitor of protein biosynthesis), cycloheximide and MG132 (proteasome-inhibitor) or neither. After 36 h cells were lysed and 50 ug of protein were blotted and hybridization performed with anti-myc antibody.

Western blot analysis indicated that co-transfection of FOXL2 and PIAS1, or UBC9, or SUMO-1 significantly increased the protein levels of FOXL2 and of sumoylated FOXL2, as revealed by quantification of the FOXL2-SUMO-1 band by densitometric analysis (Fig. 2A). The interaction of FOXL2 with PIAS1, UBC9 and/or SUMO-1 might thus lead to stabilization of the 50 KDa FOXL2 polypeptide in a dose-sensitive manner (Fig. 2A, B). These bands were not detected or strongly reduced when the sample was not treated with NEM (Fig. 2A).

According to Protparam software (http://www.expasy.ch/tools/protparam.html) the estimated half-life for FOXL2 in mammalian reticulocytes is around 30 hours, and the instability index is computed to be 66.04, classifying the protein as unstable. We confirmed the prediction of FOXL2 half-life by the addition of cycloheximide, an inhibitor of protein biosynthesis in eukaryotic organisms, in a time-course experiment, followed by immunoblotting with anti-myc antibody (Fig. 2C). The treatment with both cycloheximide and MG132, a proteasome inhibitor, showed that the stability of FOXL2 is dependent on the co-transfection with SUMO-1. Furthermore it is clear that FOXL2 stability is not dependent on MG132 treatment, suggesting that FOXL2 degradation is not mediated by proteasomes. To check if FOXL2 is post-translationally modified by ubiquitin, which targets proteins to be degradated by proteasome, we performed a Foxl2 immunoprecipitation in α-T31 cell lines, followed by western blot with anti-ubiquitin antibody. This assay shows that Foxl2 is not conjugated with ubiquitin (data not shown). This is consistent with reports indicating that ubiquitin-mediated degradation is usually important for proteins with a half-life of 3–5 hours, while for proteins with half-lives longer than 30 hours degradation is linked to lysosomes [12]. These results suggest a role of sumoylation in regulating FOXL2 stability. It seems likely, from other cases that have been studied in detail, that sumoylation can directly affect protein stability, but a direct effect has not been proven in this case.

### Foxl2, Pias1, Ubc9 and Sumo-1 Are Co-Localized in the Mouse Ovary

**Figure 3:**
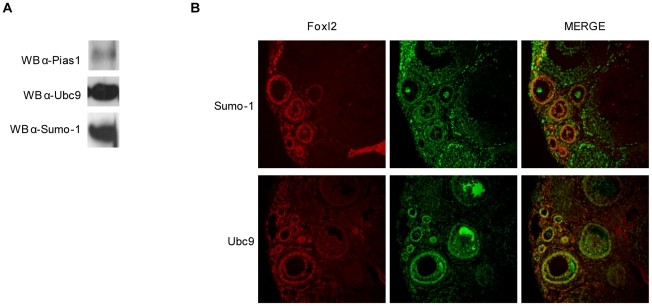
Foxl2, Sumo-1 and Ubc9 are expressed in mouse ovary. *A.* Pias1, Ubc9 and Sumo-1 are expressed in 4 week mouse ovaries: 4-week old mouse ovaries were lysed and analysed by immunoblotting with anti- Pias1, Ubc9, and Sumo-1 specific antibodies. 50 ug of protein were loaded in each lane. *B.* Co-Localization of Foxl2, Sumo-1 and Ubc9 in 4 week-old mouse ovary: Hybridization with anti-Foxl2 (red) and anti-Ubc9 (green) or anti-Sumo-1 (green) antibodies. Yellow spots in the 40× magnification show the co-localization of Foxl2 with Ubc9 and Sumo-1, especially in primordial and primary follicle granulosa cells. Immunofluorescence analysis was performed using a Leica DMIRE2-TCS-SL Confocal Laser Scanning microscope (from 488 to 633 excitation wavelength).

Recently Shao et al [13], [14] reported that Sumo-1, based on immunohistochemical analyses, is expressed in both healthy and atretic mouse granulosa cells *in vivo*, suggesting that Sumo-1 may function both during follicular development and atresia. We performed western blotting on protein extracts and immunofluorescence on sections of 4-week mouse ovaries, using specific antibodies for Foxl2, Pias1, Ubc9 and Sumo-1. The signal for Pias1 by immunofluorescence was low and visible only in western blotting. The other proteins were detected both by western blotting (Fig. 3A) and immunofluorescence (Fig. 3B). Foxl2, expressed in the granulosa cells of ovarian follicles at different stages of development, was co-localized with both Sumo-1 and Ubc9 (Fig. 3B).

### Identification of Sumoylation Sites

**Figure 4:**
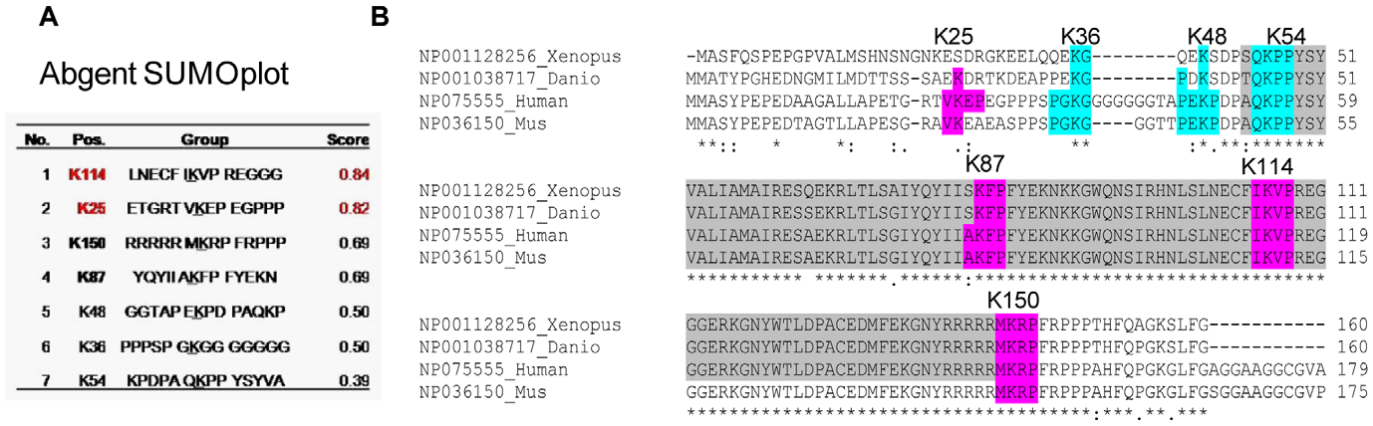
Sumoylation sites prediction. *A.* The SUMOplot™ Analysis Program (http://www.abgent.com/sumoplot.html) was used to predict and score sumoylation sites within the FOXL2 protein (upper panel). *B.* Sequence alignment of the FOXL2 region surrounding the putative sumoylation sites using the ClustalW2 Web based tool (right panel). In grey is highlighted the forkhead domain. In pink the SUMO consensus sequences K25, K87, K114, K150, with higher score, in blue those with lower scores (K36, K48, K54).

More than two-thirds of the known SUMO substrate proteins have a consensus sumoylation motif ΨkxE, where Ψ represents a hydrophobic amino acid, X represents any amino acid and K is the leucine residue where SUMO-1 is covalently conjugated [8]. We used a computational method “The SUMOplot™ Analysis Program (http://www.abgent.com/sumoplot.html)” to predict and score potential sumoylation sites within the FOXL2 protein (Fig. 4A). The program identified 7 putative sumoylation sites at positions K25, K36, K48, K54, K87, K114, and K150, all of which lie in and around the forkhead domain. The sites containing lysine residues 54, 87, 114 and 150 are strongly conserved across many phyla, whereas those containing the lysine residues 25, 36 and 48 are found only in mammals. The alignment of FOXL2 amino acid sequences retrieved from the Ensembl database (http://www.ensembl.org), was done by the ClustalW2 Web based tool (http://www.clustal.org) (Fig. 4B).

**Figure 5:**
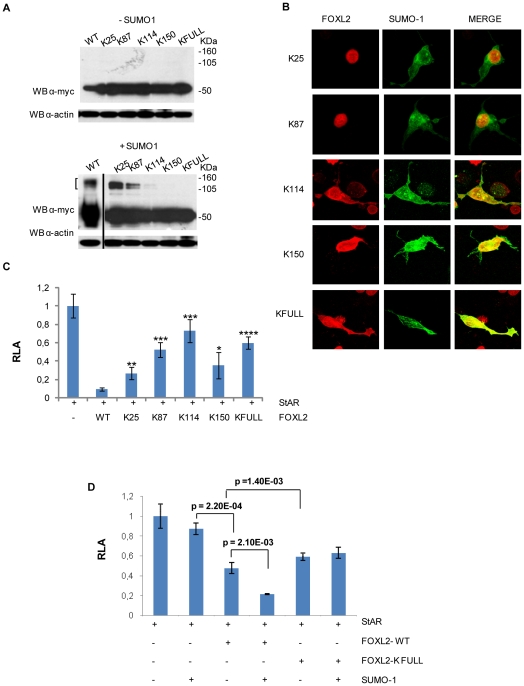
Identification and characterization of putative FOXL2 sumoylation sites. *A.* Mutations of K114, K150 and KFULL results in a strong reduction of the largest sumoylated band: COS 7 cells were transfected with pCRUZ-myc-FOXL2 wild type or in the mutated forms, with and without pCRUZ-HA-SUMO-1. 48 h after transfection cells were lysed and 50 ug of protein were analysed by western blotting with anti-myc antibody. The largest sumoylated band is absent in K114, K150, and K-FULL. *B.* Mutation of K114 or K150 affects FOXL2 cellular localization: pCRUZ-myc-FOXL2 (WT, K25R, K87R, K114R, K150R, FULL) and pCRUZ- HA-SUMO-1 were co-transfected in COS-7 cells. The intracellular distribution of FOXL2 (red) and SUMO-1 (green) was detected by indirect immunofluorescence with mouse anti-myc and rabbit anti-HA primary antibodies and Alexa Fluor 633 anti-mouse and Alexa Fluor 488 anti-rabbit secondary antibodies. Yellow spots show the overlap (co-localization) of the two signals. *C., D.* Mutation of putative sumoylation sites results in changes of FOXL2 transcriptional activity: (C) COS-7 cells were co-transfected with pGL3-StAR luciferase reporter vector and wild-type or mutated FOXL2. Reporter Luciferase activity expressed from the StAR promoter was measured. Luciferase activity is reported as relative activity. It is the mean of at least 4 independent experiments, normalized to the reporter gene *Renilla*, encoded by a pTRLK vector, used as an internal control, and compared to promoter activity alone. Significance was estimated using Student's t-Test, (*) p<0.01, (**)<0.001, (***)<0.0001, (****)<10^−7^ *(D)* COS-7 cells were co-transfected with pGL3-StAR luciferase reporter vector, and pCRUZ-myc-FOXL2 wild-type or pCRUZ-myc-FOXL2-KFULL, with or without pCRUZ-HA-SUMO-1. The mutation of all 4 putative sumoylation sites (FOXL2-KFULL) leads to a level of FOXL2 inhibition independent of the addition of SUMO-1, whereas wild-type mediated inhibition is increased by SUMO-1. Significance was estimated using Student's t-Test and reported in the chart.

We created FOXL2 mutants in which the lysines of the higher score putative sumoylation sytes (K25, K87, K114, K150) were changed to arginine by site-directed mutagenesis, alone or all together (KFULL). After transfection in COS-7 cells of mutant forms of myc-FOXL2 with and without SUMO-1, lysates were immunoblotted with anti-myc antibody. The results show that putative sumoylated band(s) appear in K25 and K87 mutants, but are strongly reduced/abrogated in K114, K150, and FOXL2-KFULL (Fig. 5A).

### Sumoylation Affects FOXL2 Localization in COS-7 Cells

To assess any effect of SUMO modification on the subnuclear localization of FOXL2, we transiently co-transfected COS-7 cells with myc-FOXL2 wild type (WT) or mutated forms and HA-SUMO-1 and then examined localization in confocal microscopy by immunofluorescence. Anti-myc antibody showed nuclear localization of FOXL2 WT; anti-HA antibody revealed that both FOXL2 and SUMO-1 at least partially co-localized in the nucleus (Fig. 1F). FOXL2-K25R or FOXL2-K87R did not change the nuclear co-localization (Fig. 5B). By contrast, when K114 or K150 were individually mutated – or when all 4 possible sumoylation sites were mutated (FOXL2-KFULL), a considerable fraction of FOXL2 became cytoplasmic, in addition to some in the nucleus (Fig. 5B).

### Sumoylation Regulates FOXL2 Transcriptional Activity

Thus far, the best characterized FOXL2 target genes are involved in steroidogenesis: the gonadotropin-releasing hormone receptor (GnRHr), the alpha subunit of the gonadotropins (Cga), and the steroidogenic acute regulatory protein (StAR) [15]–[17]. In particular, FOXL2 represses the expression of the *StAR* promoter, which harbours 15 potential forkhead-responsive recognition motifs, in CHO (Chinese hamster ovarian) cells [17]. To assess any effects of FOXL2 sumoylation on transcriptional activity, we carried out a series of transactivation assays with luciferase as a reporter gene, driven by the 972 bp promoter of the human *StAR* gene in the presence or absence of wild type FOXL2 and mutated forms, each of them alone or along with SUMO-1. Studies were carried out both in CHO (data not shown) and COS-7 cells. Confirming earlier findings [17], FOXL2 sharply inhibited *StAR* transcription (Fig. 5C). We further showed that sumoylation increased the extent of repression (Fig. 5D). All single FOXL2 mutants K25R, K87R, K114R and K150R have significantly decreased activity on the *StAR* promoter, and the effect is confirmed with the FOXL2-KFULL mutated form (Fig. 5C). The reduction in repression activity of K25 and K87, since do not seem to be sumoylation sites, might be due to a different mechanism, such as alteration in DNA/protein binding.

**Figure 6:**
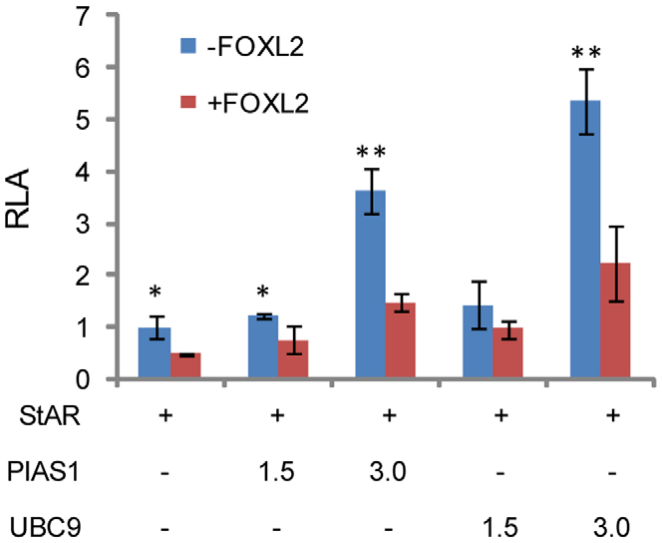
PIAS1 and UBC9 in contrast to FOXL2, activate *StAR* promoter. COS-7 cells were co-transfected with pGL3-StAR luciferase reporter vector, and increasing amounts (1.5, 3 µg) of PIAS1 or UBC9, with or without FOXL2 (1 µg). Relative luciferase activity is compared to that of promoter activity alone. Luciferase activity is reported as relative activity, as the mean of at least 4 independent experiments, normalized to the reporter gene *Renilla*, encoded by pTRLK vector, used as an internal control, and compared to promoter activity alone. Statistical significance was estimated with Student's t-Test, p-values <0.05 are significative, (*) p<0.01, (**) p<0.001.

All the data collected so far suggest that the increase in the repressing activity of the FOXL2 in presence of SUMO-1 is likely due in large part to stabilization of FOXL2, although increased binding of FOXL2 to DNA might play a role, as suggested for SOX9 [6]. Additional transactivation assays were carried out co-transfecting FOXL2 and increasing amounts of PIAS1 and UBC9 in COS-7 cells, to test whether FOXL2 transcriptional activity, like that of SOX9, is affected by PIAS1 in both SUMO-ligase-dependent and independent manners [7] (Fig. 6). The responses of the *StAR* promoter construct to PIAS1, UBC9 and FOXL2 expression showed that FOXL2 was clearly a repressor of the *StAR* promoter, whereas PIAS1 and UBC9 alone effectively stimulated the *StAR* promoter in a dose-dependent manner (Fig. 6). Furthermore, these results imply that the effects of FOXL2 on the *StAR* promoter are strongly modulated by interactions with other transcriptional modulators, in this case PIAS1 and UBC9.

## Discussion

As a forkhead transcription factor with a primary role in follicle and eyelid development, the importance of FOXL2 in ovarian development and function is unquestioned, but its expression is under complex transcriptional control [18], and it has not been known how its action might be modulated during gonadal development. It is now clear that sumoylation is a vital control of FOXL2, and it shows an intriguingly symmetrical action in the yin and yang of FOXL2 and SOX9 in the determination of sex and gonadal development.

Sumoylation affects many processes (see Introduction), and has been suggested to be involved in reproductive functions [19], [20]. SUMO-1 protein is widely expressed in various tissues, and in particular it has already been reported in the entire granulosa cell population of all follicles, from primordial to early antral stage, in ovaries of immature mice [14]. Stimulation of LH receptors in granulosa cells results in down-regulation of SUMO-1 expression with concomitant induction of ovulation [14]. SUMO-1 has also been localized in male germ cells and somatic cells of mouse and rat testes [19] as well as in human male germ and somatic cells [20]. A role of SUMO-1 in modulating steroid action and potentially diverse functions during spermatogenesis has been suggested [20], presumably driven by SOX9.

In this study, we report that the modification of FOXL2 by SUMO-1 in transiently transfected monkey kidney cell lines has profound effects on its stability and cellular localization, with an apparent resultant effect on transcriptional activity. We have also shown comparable sumoylation for endogenous mouse Foxl2 (Fig. 1D, E). It seems very likely that endogenous human FOXL2 is also comparably sumoylated, though that has not been directly demonstrated.

As for the sites of substitution, FOXL2 sumoylation has been recently reported [21], [22]. Kuo et al [22] indicate K25 as the only sumoylation site. Here we show that in fact the four predicted sumoylated sites with the highest score according to SUMOplot prediction are all likely to be involved in FOXL2 function, because they affect localization (K114R, K150R, KFULL); sumoylation (K114R, K150R, KFULL); and transcriptional activity (all the four mutations singly and all together). In our results, a sumoylated band is present in K25 and K87 FOXL2 mutants, while it is almost completely lost in K114R, K150R and KFULL. It may be that sumoylation can affect different sites with different efficiency in different cellular environment. Although FOXL2 has 4 sumoylation sites, a mutation in K114 or K150 did not reduce the molecular weight but only decreased/abrogated the intensity of the 105–160 KDa band(s). These results suggest that FOXL2 is more likely modified at a major FOXL2 sumoylation site with a poly-SUMO-1 chain rather than being mono-sumoylated at 4 different lysines.

Taking into account the comparable effects on both FOXL2 and SOX9, sumoylation may be a critical regulator of a wide variety of HOX transcription factors. In the gonad in particular, the parallelism of sumoylation and its effects on FOXL2 and its mutual antagonist SOX9 in differentiation is striking. As with SOX9 [6], [7], PIAS1 interacts directly with FOXL2 in two-hybrid assays and co-immunoprecipitation, enhancing sumoylation and at least partially inhibiting degradation by the proteasome pathway. The analogy extends to effects on transcriptional activity. In COS-7 cells, PIAS1 stimulated the SOX9-dependent transcriptional activity of a *Col2a1* promoter-enhancer reporter, and this increase in reporter activity was followed by an increase in the cellular levels of SOX9 [6]. In our study PIAS1 and UBC9 are *StAR* promoter activators, and showed an effect opposite to FOXL2, which acts as a repressor of the *StAR* promoter.

It is important to note that the effects of sumoylation – especially because it is balanced to some extent by de-sumoylation – are not all-or-none, but rather provide a finer modulation of the levels and localization of the transcription factors. Thus, although SUMO modulation changes the effective level of FOXL2 or SOX9 activity, there is no evidence that sumoylation might differentially affect one of the transcription factors; and it therefore remains unclear if it critically affects the outcome of FOXL2/SOX9 competition in sex determination. As we have discussed elsewhere, that competition is certainly governed by other factors as well. They include the over-riding power of the Y chromosome gene SRY in determining SOX9 expression and testis determination, as well as the timing of SRY/SOX9 vs. FOXL2 expression [23]. On the other hand, quantitative changes in the level of their activity can be consequential and can in fact be determinative, as seen by the profound effects and partial sex reversal shown in individuals heterozygous and thereby haploinsufficient for FOXL2 or SOX9.
